# The Role of microRNAs in the Cisplatin- and Radio-Resistance of Cervical Cancer

**DOI:** 10.3390/cancers13051168

**Published:** 2021-03-09

**Authors:** Rina Masadah, Syahrul Rauf, Muhammad Yogi Pratama, Claudio Tiribelli, Devis Pascut

**Affiliations:** 1 Department of Pathology Anatomy, Hasanuddin University, Makassar 90245, Indonesia; yogi.pratama@fegato.it; 2 Department of Obstetrics and Gynecology, Hasanuddin University, Makassar 90245, Indonesia; syahrulrauf@med.unhas.ac.id; 3 Liver Research Center, Fondazione Italiana Fegato—ONLUS, Basovizza, 34149 Trieste, Italy; ctliver@fegato.it (C.T.); devis.pascut@fegato.it (D.P.)

**Keywords:** cervical cancer, cisplatin, microRNA, drug resistance, radiotherapy, cisplatin resistance

## Abstract

Cervical cancer is the fourth leading cause of cancer-related death among women worldwide. The chemotherapeutical agent cisplatin, a small platinum-based compound, is considered as the standard therapy for locally advanced cervical cancer or recurrent cancers, sometimes in combination with radiotherapy or other drugs. However, drug resistance and radio-resistance phenomena could reduce the life expectancy of cervical cancer patients. Resistance mechanisms are complex and often involve multiple cellular pathways in which microRNAs (miRNAs) play a fundamental role. miRNAs are a class of endogenous non-coding small RNAs responsible for post-transcriptional gene regulation. Convincing evidence demonstrates that several deregulated miRNAs are important regulators in the onset of drug and radioresistance in cervical cancer, thus underlying their potential applications in a clinical setting. In this review, we summarized the mechanisms by which miRNAs affect both cisplatin and radioresistance in cervical cancer. We also described the regulatory loops between miRNAs and lncRNAs promoting drug resistance. Besides, we reported evidence for the role of miRNAs in sensitizing cancer cells to cisplatin-based chemotherapy, and provided some suggestions for the development of new combined therapies for cervical cancer.

## 1. Introduction

Cervical cancer represents the fourth most common cancer in females worldwide [1] with 570,000 women diagnosed each year (www.who.int, accessed on 10 January 2021). Almost all cervical cancers arise in a small anatomic area known as the cervical transformation zone (TZ) that develops between the secretory columnar epithelium of the endocervix and the stratified squamous epithelium of the ectocervix [2]. The lesion can be categorized into two subtypes, squamous cell carcinomas (SCC around 80%) and adenocarcinomas (around 5–20%) [3,4].

Despite several risk factors having been identified, such as early age of initial sexual encounter or age of initial pregnancy, smoking status, long use of oral contraceptive pills, weak immune systems, family history, and genetic variation, human papillomavirus (HPV) infection represents the main cause for the development of cervical cancer contributing to 70% of cervical cancer worldwide [5,6,7].

### Cervical Cancer Treatments and Cisplatin Resistance

The guidelines for the treatment of cervical cancer are provided by the International Federation of Gynecology and Obstetrics (FIGO) which is also suggesting a clinical staging system (FIGO staging system) [8]. Early-stage cervical cancers, in which surgery and primary radiotherapy represent the eligible treatments, have a decent prognosis, with almost 80% of the subjects reaching a 5-year survival [9,10]. Almost twenty years ago, the National Cancer Institute Alert demonstrated the superiority of cisplatin-containing concurrent chemoradiotherapy for the treatment of advanced cervical cancer [11]. Based on this evidence, over time the chemotherapeutical agent cisplatin, a small platinum-based compound, became the worldwide standard for chemotherapy [12,13]. Despite the fact that studies have demonstrated the value of the use of neoadjuvant cisplatin-based chemotherapy (NACT) to treat FIGO stage IB2, IIA2, and IIB, compared to the primary surgery treatment (PST) [14], cisplatin has been considered the primary chemotherapy for locally advanced cervical cancer (LACC) or recurrent cancers, even in combination with radiotherapy or other drugs [15,16]. However, it should be taken into consideration that the majority of patients treated for persistent and/or recurrent cervical cancer represent previous chemo- or chemoradiation failures. Thus, for some patients, the establishment of certain molecular mechanisms impairing the response to cisplatin might have a significant role in determining a poor prognosis. Since forty years ago, studies have assessed the response rates to cisplatin, varying from 18% to 50% with doses ranging from 50 to 100 mg/m^2^ every 3 weeks [15,17,18]. More recently, in a phase III randomized trial evaluating the response to first-line chemotherapy in patients with stage IVB, recurrent, or persistent cervical cancer [19], a 30% (14.0–27.5, 95%CI) overall response rate to cisplatin was observed. Importantly 6 out of 149 patients had a complete response to cisplatin-based therapy [19]. Thus, low response rates and resistance mechanisms still represent an unsolved issue in clinics. Data from three Gynecologic Oncology Group (GOG) phase III studies (protocols 110, 149, and 169), evaluating the effect of single-agent cisplatin compared to cisplatin plus ifosfamide, cisplatin plus paclitaxel, and cisplatin plus topotecan, identified 5 factors independently associated with the response to cisplatin therapy [20]. Race, performance status (PS), site of disease, prior radiosensitizer, and the interval between diagnosis and 1st recurrence, have been included in a prognostic index able to classify patients into three risk groups. Given the similar weights and no interactions between factors, an index based on the total number of risk factors was derived (low risk: 0–1 factor, moderate risk: 2–3 factors, high risk: 4–5 factors). Patients with more risk factors (*n* = 4–5) are estimated to have a response rate of 13% and a median disease-free survival and overall survival of 2.8 months and 5.5 months, respectively [20]. Despite the evidence for clinical and phenotypical variables able to classify patients according to the response to treatment, the comprehension of the molecular mechanisms in determining drug and radioresistance could promote the advance to a personalized medicine approach, in which molecular biomarker can find a relevant decisional role.

## 2. Molecular Mechanisms Associated with Cisplatin Resistance: The Role of microRNAs

The molecular mechanisms responsible for the cisplatin antitumor activity involve multiple intertwined pathways. DNA binding and crosslinking are some of the major cisplatin-induced DNA damages. When the amount of DNA damage is no longer sustainable, cells undergo apoptosis [21]. Besides, cisplatin triggers ROS production from damaged mitochondria, destroys lysosomes which in turn release lysosomal proteases, degrades endoplasmic reticulum determining impairment in calcium storage and misfolded proteins, and it can bind to membrane-bound Na^+^/H^+^ exchanger protein (NHE), Thioredoxin reductase (TrxR), tubulin, and other proteins, impairing their function [22,23].

Despite the multitude of the mechanisms by which cisplatin damages cancer cells, resistance, either intrinsic or acquired, may develop and seriously compromise the efficacy of the drug in clinical practice. Over the past three decades, great efforts were dedicated to characterize the molecular mechanisms underlying cisplatin resistance in cervical cancer [13]. Cisplatin resistance phenomena occur through the reduction in the intracellular accumulation, increased activation or modifications in the DNA damage repair system, alterations in the apoptotic pathway, activation of epithelial–mesenchymal transition (EMT), and alteration in DNA methylation [13,23]. It has been demonstrated that cancer stem cells show high resistance to cisplatin [24]. They express a large number of multidrug resistance transporters such as multidrug resistance protein 1 (MDR1/P-gp/ABCB1) and mitoxantrone resistance protein (MXR)/cancer breast resistance protein-1 (BCRP-1)/ATP Binding Cassette Subfamily G Member 2 (ABCG2) [24], responsible for the drug efflux. Moreover, high activity of aldehyde dehydrogenase (ALDH), a marker for cervical cancer stem cells, was associated with increased resistance to cisplatin in cervical cancer cells [25]. In all these pathways, the regulatory small non-coding RNAs (sncRNAs), microRNA (miRNAs), play a pivotal role in determining cisplatin resistance [26,27,28,29,30].

miRNAs represent an evolutionarily conserved class of small, endogenous non-coding RNA molecules that modulate the expression of different target mRNAs [31]. The binding of the miRNA with the 3′ untranslated region (3′UTR) on the mRNA target can determine the complete degradation of the mRNA molecule, in the case of full miRNA/mRNA complementarity, or the prevention of translation in case of partial complementarity. Importantly, each miRNA can target several types of mRNA molecules and each mRNA may contain multiple binding sites for different miRNAs, thus determining a complex, but at the same time, finely controlled, regulatory network [32].

Since their discovery in 1993 [33], miRNAs have attracted wide attention owing to their unique functional role in cells. However, with the first reported evidence of the involvement of miRNAs in human cancer, derived from studies on chronic lymphocytic leukemia (CLL) [34], miRNAs became the widely studied class of sncRNAs in the field.

Many recent studies have demonstrated that the deregulation of miRNAs contributes to cervical cancer development, where they modulate tumor growth, invasion, angiogenesis, immune evasion, and drug resistance [35,36].

The earliest studies about the role of miRNA in cervical cancer drug resistance date back to 2012 when miR-145 was identified as a part of a p53-miR-145 axis regulating the IGF signaling pathway responsible for mitomycin resistance in cervical cancer [37]. More recently, several studies contributed to elucidate the role of miRNAs in cisplatin resistance in cervical cancer. A recent study from Fekete and colleagues [38] investigated the potential role of miRNAs as predictors of platinum-based chemotherapy for squamous cell cervical cancer. By using data retrieved from the TCGA repositories they identified three miRNAs (miR-342, miR-378c, and miR-155) able to distinguish responder patients (*n* = 78) from non-responders (*n* = 16) with an AUC higher than 0.8 [38], thus suggesting a possible role for these miRNAs in platinum resistance mechanisms in cervical cancer.

### 2.1. Up-Regulated miRNAs Determine Resistance to Cisplatin in Cervical Cancer

**Figure 1:**
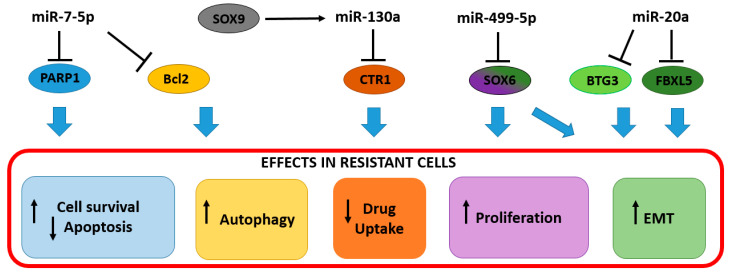
Up-regulated miRNAs in drug resistant cancer cells and tissues. The increased levels of oncogenic miRNAs involved in cisplatin resistance inhibit targets involved in several cell pathways such as apoptosis, drug transport, proliferation EMT. Blue arrows indicate the cellular effect due to the inhibition of the miRNA target. EMT (Epithelial–Mesenchymal Transition). Color gradation indicate the multiple role of the miRNA target.

Due to their role as post-transcriptional gene regulators, miRNAs play also a role in the modulation of cisplatin resistance. The up-regulation of miR-7-5p in cancer tissues was associated with therapy failure [26]. In vitro investigations found miR-7-5p increased in HeLa and SiHa cervical cancer cells with acquired cisplatin resistance. In those cells, miR-7-5p exerts a dual function by targeting both poly ADP-ribose polymerase 1 (*PARP-1*) and B-cell lymphoma 2 (*BCL2)* (Table 1). From one side, the repression of *PARP-1* prevents resistant cells to undergo apoptosis, on the other side, the downregulation of *BCL2* ensures energy availability through an autophagic process [26]. In non-resistant cells, the high-energy demand for the DNA repair mechanism leads to cell dysfunction, followed by apoptosis. The up-regulation of miR-7-5p and the consequent *PARP-1* downregulation, not only avoid apoptosis but also contribute to moderate the DNA repair mechanisms, as shown by the slight increase of the sensitive DNA damage marker, the phosphorylated H2A histone family member X (γH2AX), in resistant cells, compared to the parental controls [26]. This ensures the maintenance of a moderate level of DNA repair, which, together with the apoptosis inhibition and autophagy stimulation, fosters resistant cell survival, sustaining the energy demand (Figure 1).

Another example of the dual role of miRNAs in cisplatin resistance came from the studies of Feng C. and colleagues [39] in which a SOX9/miR-130a/CTR1 axis was found to participate in drug resistance. In the proposed model, SRY-Box Transcription Factor 9 (SOX9) was able to stimulate the expression of miR-130a in both cisplatin-resistant cells and tissues. The overexpression of miR-130a resulted in the reduction of the Copper Transporter 1 (CTR1 or hCtr1, encoded by *SLC31A1 gene*), a copper influx transporter required for high-affinity copper (probably reduced Cu I) transport into the cell, responsible for the cisplatin accumulation in tumor cells [51] (Table 1). Thus, the reduced cisplatin uptake contributed to HeLa and CaSki cell survival and proliferation, together with the inhibition of Phosphatase and Tensin Homolog (*PTEN),* the other known miR-130a target [52] (Figure 1).

The acquisition of EMT features represents a key contributor to chemoresistance in cervical cancer, thus the expression of the EMT repressors F-Box and Leucine Rich Repeat Protein 5 (*FBXL5*) and BTG Anti-Proliferation Factor 3 (*BTG3*) is retained low in cancer tissues and cisplatin-resistant cells. It has been shown that miR-20a has an important role in the downregulation of *FBXL*5 and *BTG3* in HeLa cells, thus fostering the resistant phenotype [27] (Table 1 and Figure 1). Importantly, the high miR-20a expression and the correspondent low *FBXL5* and *BTG3* expression in cancer tissues correlate with the poor prognosis of the patients in a Kaplan–Meier survival analysis [27].

In experiments conducted by Chen and colleagues (2020), the miR-499a-5p transfection, increased migration, invasion, and resistance to cisplatin both in cells and mouse models [44]. In this model, miR-499a-5p was shown to target SRY-Box Transcription Factor 6 (*SOX6*) an important tumor suppressor playing roles in cell proliferation, differentiation, and cell fate determination (Table 1 and Figure 1). Despite this evidence, recent studies showed the downregulation of miR-499a-5p in cervical cancer tissues compared to adjacent normal tissues [53], thus the role of this miRNA in cervical cancer should be further clarified.

### 2.2. The Downregulation of Cellular miRNAs Promotes Cisplatin Resistance in Cervical Cancer

**Figure 2:**
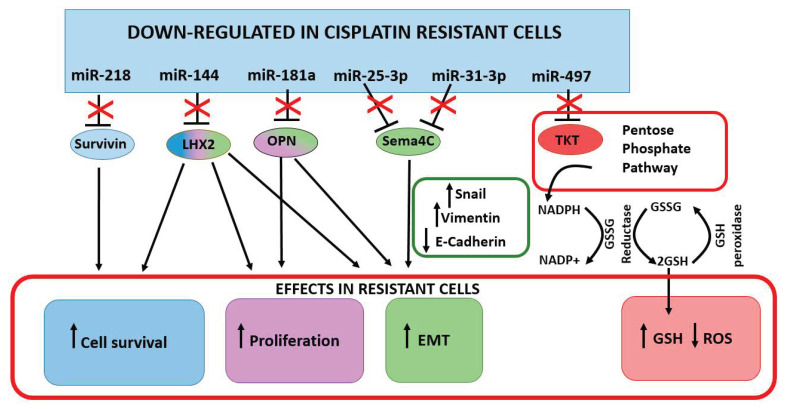
Down-regulated miRNA in drug resistant cancer cells and tissues. The decreased expression of oncosuppressor miRNAs promote cisplatin resistance. Two miRNA, miR-25-3p and miR-31-3p, have been shown to target *SEMA4C* (Semaphorin 4C), although in different experimental models. By inhibiting *TKT* (transketolase), miR-497 prevents the availability of NADPH used for the production of GSH, an important antioxidant molecule. High levels of GSH are fundamental for the ROS detoxification, which are abundantly produced in cancer cells. Color gradation indicate the multiple role of the miRNA target.

A growing body of evidence suggested miR-218 as significantly downregulated in human cancer tissues compared to the adjacent non-cancerous tissues [54,55], including cervical cancer [56], where it plays a role as a tumor-suppressor miRNA. MiR-218 was found to be significantly downregulated in cisplatin-resistant HeLa and SiHa cells compared to parental controls reaching a 60% decrease in both cell lines [40] (Table 1). Survivin, one of the well-known inhibitors of apoptosis, was identified as a miR-218 target, thus the suppression of this miR in HeLa and SiHa cells ensures cell survival through a survivin-dependent mechanism [40]. Indeed, the restoration of miR-218 in cells determines a remarkable decrease in cell proliferation and contributes to cell chemosensitivity toward cisplatin [57] (Figure 2).

Another miRNA downregulated in cisplatin-resistant cells is miR-144. In resistant HeLa and SiHa cells, Shi and colleagues [41] observed a 50% downregulation of miR-144 compared to the parental controls, and this decrease was negatively correlated with the increased expression of LIM Homeobox 2 (*LHX2*) (Table 1). LXH2 is a transcription factor implicated in multiple biological processes. It promotes tumor growth and metastasis [58,59]. Recent studies found LHX2 elevated in cervical cancer tissues compared to the normal controls [60]. The transient up-regulation of miR-144 significantly reduced *LHX2* expression inducing growth inhibition, increased cell apoptosis rate, and a reduction of migration and invasion of resistant cells [41]. The gain of function of LHX2 partly abolished the negative regulation of miR-144 in growth, migration and invasion, suggesting the importance of the LHX2 in the acquired cisplatin resistance of cells [41] (Figure 2).

MiR-25-3p was downregulated in HeLa and Caski cells resistant to cisplatin. Semaphorin 4C (*SEMA4C*), which belongs to the Semaphorins family of secreted and membrane-bound proteins, was identified as a miR-25-3p target [28]. Semaphorins were first identified in the nervous system as axonal molecules, however, they have several functions, not only in neurons but also in immune cells and cancer [61,62]. In tumors, SEMA4C is produced by tumor-associated lymphatic endothelial cells (LECs) and promotes lymphatic metastasis [63]. It has an important role in promoting EMT contributing to the acquisition of invasive properties of cervical cancer cells [28,29]. The repression of miR-25-3p ensures high levels of SEMA4C in cisplatin resistant cervical cancer cells (Table 1). However when restored, miR-25-3p not only reduces the EMT features of cancer cells, by suppressing SEMA4C, SNAIL, and Vimentin and by upregulating E-cadherin, but also sensitizes HeLa and Caski resistant cells to cisplatin [28], underlying its importance as a regulator of EMT and chemoresistance. Besides, in vivo experiments showed a 50% growth reduction of tumors generated from HeLa resistant cells stably expressing miR-25-3p injected in mouse models [28]. This effect was associated with a reversion of the EMT phenotype with upregulation of E-cadherin and a downregulation of SEMA4C and SNAIL in tumors [28]. Further proofs of the role of SEMA4C in cervical cancer can be derived from the studies of Jing L. and colleagues [29]. The downregulation of miR-31-3p in Caski cells was associated with an increase of SEMA4C and EMT features. Conversely, when the expression of miR-31-3p was restored, Caski cells showed an increased sensitivity to cisplatin compared to controls [29]. This evidence supported the association of the low miR-31-3p levels with paracervical invasion, FIGO staging, and histopathological classification. Patients with low miR-31-3p expression levels had a significantly shorter 1-year and 3-year OS rates (65 and 38%, respectively) than those with high miR-31-3p expression levels (78 and 52%, respectively) [29] (Figure 2).

Osteopontin (OPN) is a calcium-binding phosphorylated glycoprotein, which was first discovered in the bone matrix. *OPN* is an oncogene associate with cervical cancer growth and invasiveness [64,65], with roles in the regulation of drug resistance in cervical cancer [66]. *OPN* was identified as a target of miR-181a. This miRNA was found downregulated in cisplatin-resistant Caski and HeLa cells, and its expression inversely correlated with *OPN* levels [42]. When restored, miR-181 promoted cell apoptosis, reduced cell proliferation, and increased sensitivity to cisplatin by downregulating *OPN* in Caski and HeLa resistant cells [42] (Table 1 and Figure 2).

The generation of reactive oxygen species (ROS) is considered one of the cisplatin-related cytotoxicity mechanisms [67,68]. Despite antioxidant molecules are fundamental in preventing ROS-induced damages during the cell life span, once the cancer is established, high levels of antioxidant levels could hamper the cytotoxic effect of chemotherapeutic agents through detoxification and increased activity of efflux pump.

Transketolase (TKT) is an enzyme involved in the pentose phosphate pathway (PPP) [69], in which NADPH is produced by glucose-6-phosphate dehydrogenase. NADPH supports the antioxidant glutathione pathway by converting oxidized glutathione (GSSG) to reduced glutathione (GSH), which is used by glutathione peroxidase to reduce H_2_O_2_ to H_2_O. Keeping the PPP highly productive is fundamental for cancer cells since their high demand in NADPH and GSH [70]. *TKT* is frequently upregulated in cancers, including cervical cancer [70,71], which was recently identified as a miR-497 target [43]. Its downregulation contributes to cisplatin chemosensitivity due to an overall GSH decrease and ROS increase, which ultimately exposes cancer cells to oxidative stress. In HeLa and SiHa cisplatin-resistant cells, the miR-497 downregulation ensures high levels of TKT which ultimately provides cancer cells with GSH to counteract the cisplatin-induced ROS-derived cytotoxicity [43] (Table 1 and Figure 2).

### 2.3. miRNAs Enhancing the Sensitivity to Cisplatin

**Figure 3:**
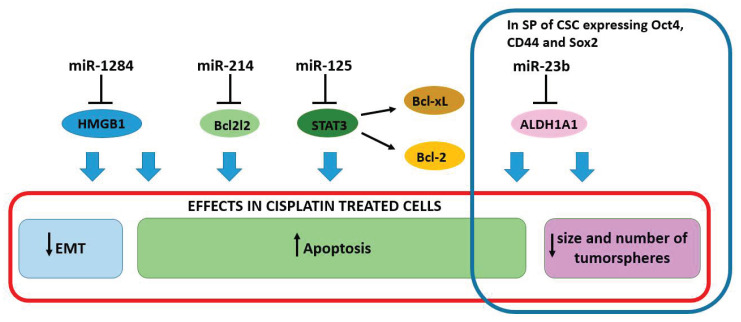
MiRNAs contribute to sensitizing cells against cisplatin. The transfection with oncosuppressor miRNAs sensitizes cells to cisplatin inducing an increase in cancer cell death. In a side population (SP) of cancer stem cells (CSC), blue rectangle, miR-23 was shown to target *ALDH1A1*, a CSC hallmark, to reduce the size and the number of tumorspheres and to sensitize cells to cisplatin. Blue arrows indicate the effect in cisplatin-treated cells due to the inhibition of the miRNA target.

A low expression of the oncosuppressor miR-1284 was observed in cervical cancer tissues and cancer cell lines. Patients with low levels of miR-1284 had a low overall survival rate, also the miR-1284 levels inversely correlated with FIGO staging, lymph node metastasis, and histological grade. The high-mobility group box-1 (*HMGB1*) was recently identified as a target of miR-1284 [45] (Table 1). *HMGB1* encodes for a nuclear protein that binds to DNA and acts as an architectural chromatin-binding factor, however, under stressful conditions, HMGB1 translocates to the cytosol with a role in promoting survival by sustaining autophagy through interactions with beclin-1 [72]. It is considered an antiapoptotic protein and studies have revealed the oncogenic role in human cancers [73,74,75]. HMGB1 played also a role in EMT and its inhibition impaired cell proliferation and invasion [45]. The same effects were observed in the presence of miR-1284. The upregulation of miR-1284 suppressed the proliferation and invasion, while promoted apoptosis. Interestingly, the miR-1284 upregulation and the consequent *HMGB1* targeting synergized with cisplatin, enhancing the sensitivity of cervical cancer cells to the drug [45]. The synergisms between miR-1284 and cisplatin were further proved by Wang and colleagues [76] that used CD59sp-conjugated miRNA-1284/cisplatin-loaded liposomes to target cervical cancer cells, showing dose-dependency and higher performance in killing HeLa cells compared to cisplatin alone. These findings open new perspectives in the use of miR-1284 as a chemosensitizer in cervical cancer (Figure 3).

Resistance to apoptosis is considered a hallmark of drug resistance. In this regard, the Bcl-2 protein family members play a relevant role in determining apoptosis in response to damage stimuli. BCL2L2, also known as BCLW, a pro-survival protein, was found up-regulated in cervical cancer tissues [46]. The level of *BCL2L2* mRNA is inversely correlated with the expression of miR-214, which is shown to directly bind to the 3′UTR region. The transfection of miR-214 into HeLa and C-33A cells induced apoptosis through activation of caspase-9 and caspase-8, and increased the cellular sensitivity to cisplatin. Indeed, the *BCL2L2* downregulation was able to increase the sensitivity to cisplatin by around 8-fold [46]. The apoptosis pathway is disrupted also at the transcriptional level with the inhibition of Signal Transducer and Activator Of Transcription 3 (STAT3), a well-characterized transcription factor that contributes to tumorigenesis and chemoresistance by promoting BCL-2 and BCL-XL expression [77] (Figure 3). In HeLa cells, miR-125 increased cisplatin sensitivity by targeting *STAT3* [47] (Table 1). The same mechanism might be responsible for paclitaxel resistance.

It is well established that cancer stem cells (CSCs) play important roles as tumor-initiating factors as well as in determining several characteristics of the malignant mass, such as tumor size, proliferation, and response to treatment; therefore, CSCs are usually associated with a poor prognosis [78]. Even though there is no universal marker, CD44, CD90, CD133, CD271, Nanog, octamer-binding transcription factor 4 (OCT4), CD44, SRY-Box Transcription Factor 2 (SOX2), epithelial cell adhesion molecules, and Aldehyde Dehydrogenase 1 Family Member A1 (ALDH1A1) are considered CSCs hallmarks. They are often used for the identification of the so-called “side population” (SP), a small subpopulation of cells within the tumor mass, having stem-like characteristics. As for other tumors, CSCs have been associated with resistance to cisplatin in cervical cancer [78]. In these cells, the repression of miR-23b is involved in stemness maintenance through ALDH1A1 upregulation [48]. Indeed, when transfected into an SP of HeLa and Caski cells, expressing OCT4, CD44, and SOX2, miR-23b dramatically reduced the size and number of tumorspheres and conferred cisplatin sensitivity to cells in vitro [48] (Figure 3). CSCs characteristics were restored with the introduction of a miR-23b inhibitor into HeLa and Caski cells that acquired a strong capacity of forming tumorspheres and a cisplatin resistance [48].

### 2.4. miRNA and lncRNA Regulatory Loops Determine Cisplatin Resistance in Cervical Cancer Cells

**Figure 4:**
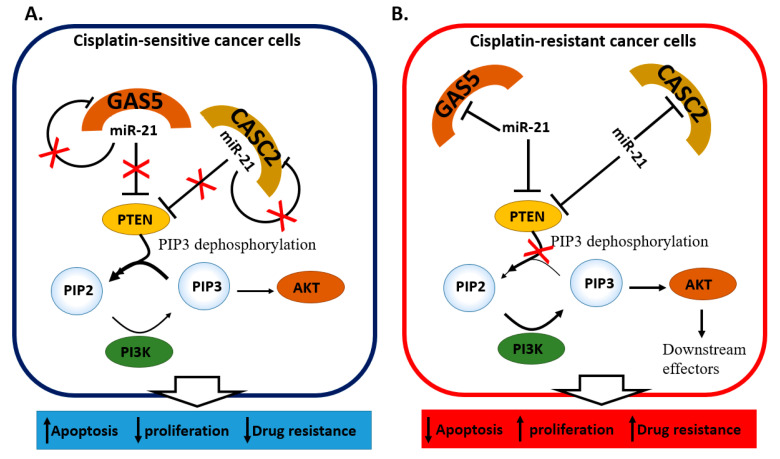

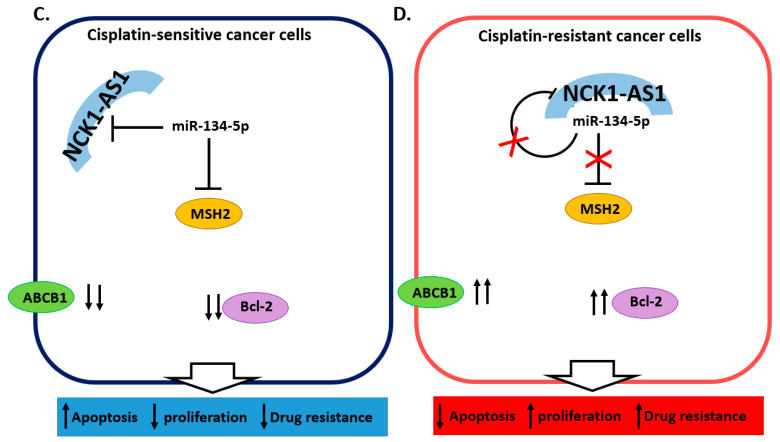
miRNA-lncRNAs regulatory loops determine cisplatin resistance in cervical cancer. In cisplatin-sensitive cells (blue rectangles) GAS5 or CASC2 lncRNAs sponge miR-21, thus PTEN dephosphorylates PIP3 into PI2, preventing AKT pathway activation, resulting in reduced proliferation, drug resistance, and increased cell apoptosis (**A**). In resistant cells (red rectangle), the downregulation of GAS5 and CASC2 cause the PTEN inhibition by miR-21 determining the PI3K/AKT pathway activation (**B**). MiR-21 can also contribute to the repression of the two lncRNAs (**A**,**B**). In cisplatin-resistant cells (red rectangle), the sponging of miR-134-5p by NCK1-AS1 leads to MSH2, ABCB1, and Bcl-2 up-regulation determining drug resistance and cell survival (**D**). In cisplatin-sensitive cells (blue rectangle), the downregulation of the lncRNA NCK1-AS1 determines the inhibition of MSH2 by miR-134-5p, associated with a decrease of ABCB1 and Bcl-2 to promote cisplatin-induced cell death (**C**).

The interaction between the miR-21 and two long non-coding RNAs, (lncRNAs) Growth arrest-specific 5 (GAS5) [49] and cancer susceptibility candidate 2 (CASC) [50], proved crucial in determining cisplatin resistance in cells. The relation between miR-21 and the two lncRNAs was hypothesized according to the inversely correlated expression observed in tumor tissues and resistant cells, with miR-21 being higher in presence of GAS5 [49] or CASC2 [50] downregulation. Further investigation from Wen and colleagues [49] identified the involvement of the PTEN/PI3K/AKT/MTOR pathway in cellular growth and cisplatin resistance. In tumoral tissues and SiHa resistant cells, the expression of miR-21 is maintained high through the downregulation of GAS5 that usually works as a miR-21 sponge. At the same time, high miR-21 levels ensure the *PTEN* repression (Table 1). Since PTEN plays an important role in suppressing the phosphatidylinositol 3-kinase (PI3K)/AKT pathway, which is associated with chemotherapeutic drug resistance in cancer cells [79,80,81], its inhibition leads to a PI3K/AKT activation with a consequent increase in cell survival and apoptosis inhibition through phosphorylating of the downstream targets [82,83,84] (Figure 4A,B). A very similar mechanism involves CASC2, which inhibited miR-21 thus promoting PTEN upregulation. In resistant HeLa and CaSki cells, the CASC2 downregulation promoted *PTEN* targeting by miR-21, the AKT phosphorylation led to resistant cell survival and proliferation [50] (Table 1). Interestingly, miR-21 can inhibit both GAS5 [49] and CASC2 [50] expression by establishing a reciprocal regulatory loop involved in cisplatin resistance (Figure 4A,B). Another group investigated the regulatory interactions among the lncRNA NCK1-AS1, miR-134-5p, and Mut S protein homolog 2 (MSH2) in cervical cancer and cisplatin resistance [30]. The expression of NCK1-AS1 was found to be almost three times higher in cervical cancer tissues compared to the adjacent non-tumoral portion and inversely correlating with the expression of miR-134-5p. In a cellular model, NCK1-AS1 was shown to competitively bind to miR-134-5p. Since miR-134-5p may reduce cisplatin resistance in HeLa cells by targeting *MSH2* (Table 1), NCK1-AS1 assumes an important role as miR-134-5p sponge, promoting tumor cell growth and drug resistance through the up-regulation of B-cell lymphoma 2 (BCL-2) and ATP Binding Cassette Subfamily B Member 1 (ABCB1) [30] (Figure 4C,D).

### 2.5. miRNAs Contributing to Radioresistance in Cervical Cancer

The combination of cisplatin-based chemotherapy with radiotherapy is a widely used therapy option for locally advanced cervical cancer (LACC) or recurrent cancer [15,16]. The addition of chemotherapy to radiation has improved local tumor control, disease-free survival rate, and overall survival in cervical tumors [85], however, as for chemotherapy, miRNAs have been found to play a significant role in promoting the cancer cell survival to radiation.

Even though there are a few reports regarding the role of miRNAs in altering the response to radiotherapy in cervical cancer, recent studies, performed in cervical cancer cell lines (SiHa cells) or tissues, have elucidated some of the mechanisms of radioresistance involving miRNAs. MiR-106b and miR-181a for example were reported as inducers of radio-resistance in cervical cancer cells [86,87]. High miR-106b expression was associated with radio-resistant tissues and cells. The inhibition of Immediate Early Response 3 (*IER3*), a mediator of apoptosis [86,88], by miR-106b increased the fraction of surviving radio-resistant ME180 cells. Further, it was described that the lncRNA GAS5, which was previously reported to influence radiosensitivity of head and neck cancer [89], also participates in the radiosensitivity of cervical cancer cell lines by down-regulating miR-106b [86]. GAS5 acted as a molecular sponge of miR-106b, thus its down-regulation in resistant cells ensures the retention of high levels of mir-106b. Indeed, when overexpressed, GAS5 enhanced the sensitivity of cervical cancer cells to treatment by up-regulating IER3 through mir-106b, proving the existence of an axis of GAS5-miR-106b-IER3 regulating radio-resistance in cancer cells [86]. This evidence proved a pivotal role of GAS5 in determining both radio- and cisplatin resistance by sponging miR-106b and miR-21, respectively (Table 1 and Table 2).

Meanwhile, in SiHa cells and in a mouse model of cervical cancer, miR-181a was described to play a critical role in modulating resistance to radiation therapy through the negative regulation of Protein Kinase C Delta (PRKCD), a pro-apoptosis gene product, further determining a decrease of the radiation-induced apoptosis and in cell fraction in the G2/M phase that is usually the most sensitive cell sub-population toward the radiation [87]. It was demonstrated that miR-181 promoted tumor cell radio-resistance by blocking the cell transition to the G2/M phase [87].

On the other hand, studies conducted in cervical cancer tissues and cell lines identified several down-regulated miRNA, such as miR-15a-3p, miR-132, miR-145, miR-499a-5p, miR-512-5p, and miR-4429, some of them, were closely related to cells apoptosis pathway. MiR-15a-3p, for example, was reported to target tumor protein D52 (*TPD52*) that positively influences the proliferation of Hela and SiHa cells, and the over-expression of miR-15a-3p in both of the cell lines promoted radiation-induced apoptosis via *TPD52* targeting [90]. Similarly, the overexpression of miR-499a-5p in both HeLa and CaSki cells after x-ray irradiation were able to enhance the radiosensitivity of cervical cancer cells, reducing colony formation, by targeting Eukaryotic translation initiation factor 4E (*eIF4AE*), a proto-oncogene that is involved in proliferation, invasion, and malignant transformation [53]. The down-regulation of miR-132 and conversely, the up-regulation of B-lymphoma Moloney murine leukemia virus insertion region-1 (*BMI-1*), that was previously identified as a key player in tumor proliferation and invasion/metastasis [91,96], was also observed in radioresistant tissues and HeLa, SiHa, and C33A cervical cancer cells [91]. It was described that the overexpression of miR-132 in resistant cells was able to decrease BMI-1 and enhance the radiosensitivity of cervical cancer cells by promoting cell apoptosis. Co-administration of exogenous miR-132 along with radiotherapy further inhibited the growth of tumors in nude mice, evidencing the potential of this miRNA as an adjuvant in radiotherapy [91] (Table 2).

Several studies have described the tumor-suppressor role of miR-145 in cervical cancer, underlining its role in sensitizing cancer cells toward radiotherapy [92,93,97]. Two miR-145 targets have been reported to be involved in the establishment of radioresistance. The DNA damage repair-associated gene Helicase-like-transcription factor (*HLTF*) was identified as a miR-145 target able to confer radio-resistance in cervical cancer cells by enhancing DNA damage repair [92]. The miR-145 overexpression was able to reduce cell viability and increase radiation-induced apoptosis by down-regulating *HLTF* (Table 2). The same effect was obtained in cells transfected with miR-145 mimic. However, in those cells, miR-145 was shown to promote radiosensitivity by targeting POU class 5 homeobox 1 (*PUO5F1*), an apoptosis inhibitor, and promoter of proliferation [93].

A newly discovered tumor-suppressor miR-4429 showed a low expression in cervical cancer cells (SiHa and HeLa) and even lower in the radio-resistant subtypes [94]. This evidence supported a possible role for miR-4429 in radiosensitizing cells. Indeed, the overexpression of miR-4429 in radio-resistant cells decreased the mRNA and protein levels of RAD51 recombinase (RAD51) (Table 2), a crucial regulator of DNA damage repair that had been previously reported as a contributor of radio and chemoresistance in cervical cancer cells [94], thus leading to increased cell death. Another interesting recent study from Zhang and colleagues (2020) observed the higher expression of miR-512-5p in both radiosensitive cervical cancer patients and cells, compared to radio-resistant counterparts [95]. Further experiments conducted in SiHa and Me180 cells showed that miR-512-5p directly targeted tumor-associated Mucin 1 (*MUC1*), an anti-apoptotic gene [95], thus promoting the apoptosis of damaged cells. In a miR-512-5p overexpressing system, a *MUC1* down-regulation was determined, thus increasing cell apoptosis and reducing cancer cell survival rate after radiotherapy [95]. All this evidence in cellular models about the role of miR-15a-3p, miR-145, miR-4429, and miR-512-5p in radiotherapy (Table 2), suggesting their potential as radio-sensitizers in cervical cancer [90,92,93,94,95].

### 2.6. miRNAs Associated with the Response to Chemoradiotherapy

Despite several experimental evidences of cellular miRNA being involved in chemo- or radioresistance in cellular models, there is still a lack of clinical studies associating tissue miRNA to treatment resistance. Besides the study from Fekete and coll., that investigated the potential role of miRNAs as predictors of platinum-based chemotherapy for squamous cell cervical cancer in the TGCA cohort [38], Pedroza-Torres and colleagues [98] identified a miRNA profile associated with the response of combined chemo and radiotherapy. Patients with locally advanced cervical cancer were treated with 5 weekly cycles of 40 mg/m^2^ of cisplatin and 67 days of radiotherapy for a total of 50–55 Gy in addition to 30 Gy of intracavitary brachytherapy. Tissue samples collected before treatments showing a high expression of miR-100-5p, miR-125a, miR-125b, miR-200a-5p, and miR-342 were found to achieve a complete response to therapy, while the high expression of miR-31-3p, and miR-3676 weas associated to a no response [98]. Both these studies indicate miR-342 as a good prognostic marker for response to treatment, supporting the possible value of this miRNA for future in vivo investigations [38,98].

Interestingly, miR-125 was shown to promote cisplatin sensitivity in vitro by targeting STAT3 [47], in line with what was observed by Pedroza-Torres in clinical specimens. On the contrary, despite miR-31-3p worked as a chemotherapy sensitizer in vitro [30], it was increased in tissues belonging to the patient not responding to chemoradiotherapy [98].

## 3. Conclusions

miRNAs are emerging as new molecular markers with possible remarkable utility in modern medicine. The clinical management of cervical cancer would benefit greatly from the understanding of the molecular pathways regulated by miRNAs, particularly to better strategize therapies. In this regard, circulating miRNA might be particularly useful. However, studies that verify the presence of miRNA involved in cisplatin and radioresistance in biofluids of cervical cancer patients are lacking. Besides, the use of miRNAs which regulate cisplatin or radioresistance may be useful as future chemo/radiosensitizers in cervical cancer. However, despite few tests, there are still obstacles to overcome before translating miRNA research into clinical trials. These include the successful delivery to neoplastic nodules, efficient penetration, and the reduction of possible off-targets effects.

## Figures and Tables

**Table 1:** MicroRNAs involved in cisplatin resistance mechanisms in cervical cancer cells.

miRNA	Expression in Cisplatin Res-Cells/Tissues	Cell Type	Target	Effect
miR-7-5p [26]	Up in resistant cells and tissues	Resistant HeLa and SiHa	*PARP-1*, *BCL2*	Inhibition of apoptosis, reduction in the DNA repair activity, increased autophagy
miR-130a [39]	Up in DDP-resistant tissue	Resistant HeLa, CaSki	*CTR1*	Proliferation, reduction in cisplatin cellular uptake
miR-20a [27]	Up in cancer tissue	HeLa	*FBXL5*, *BTG3*	Enhanced EMT phenotype, and resistance to cisplatin
miR-218 [40]	Down in resistant cell	Resistant HeLa and SiHa	Survivin	Increased survival in resistant cells, apoptosis escape mechanisms.
miR-144 [41]	Down in resistant cell	Resistant HeLa and SiHa	* LHX2 *	Cisplatin resistance, reduced apoptosis, increased migration, and invasion.
miR-25-3p [28]	Down in resistant cells	Resistant HeLa and CaSki cells	* SEMA4C *	Decreased sensitivity to cisplatin cells, enhanced EMT phenotype
miR-31-3p [29]	Down in tumor tissue	Caski	* SEMA4C *	Decreased sensitivity to cisplatin, enhanced EMT phenotype
miR-181a [42]	Down in resistant cells	Resistant HeLa and CaSki cells	* OPN *	Decreased sensitivity to cisplatin, enhanced proliferation, and resistance to apoptosis
miR-497 [43]	Down in resistant cells	Resistant HeLa and SiHa cells	* TKT *	Increased NADPH and GHS production. Hampering the ROS-dependent drug cytotoxicity
miR-499a-5p [44]	Low in HeLa High in SiHa	HeLa, SiHa	* SOX6 *	Increased migration, invasion, and resistance to cisplatin
miR-1284 [45]	Down in tumor tissue	SiHa	* HMGB1 *	Decreased sensitivity to cisplatin
miR-214 [46]	Down in cancer cells	HeLa and SiHa cells	* BCL2L2 *	Decreased sensitivity to cisplatin, resistance to apoptosis.
miR-125 [47]	Down in cancer cells	HeLa	* STAT3 *	Increased sensitivity to cisplatin
miR-23b [48]	Down in cervical cancer stem cells	HeLa, Caski	* ALDH1A1 *	Decreased sensitivity to cisplatin
miR-21 [49]	Up in DDP-resistant cells and tissue	Resistant SiHa cells	*PTEN*, *GAS5*	Cellular growth and drug resistance through the PTEN/PI3K/AKT/mTOR pathway
miR-21 [50]	Up in DDP-resistant cells and tissue	Resistant HeLa and CaSki cells	*PTEN*, *CASC2*	Cellular growth and drug resistance through the PTEN/PI3K/AKT/mTOR pathway
miR-134-5p [30]	Down in tumor tissue and resistant cells	HeLa	* MSH2 *	Decreased sensitivity to cisplatin

**Table 2:** MicroRNAs involved in radioresistance in cervical cancer.

miRNA	Expression in Radio-Resistant Cancer Cell/Tissues	Cell Type	Target	Effect
miR-106b [86]	Up in resistant cells and tissue	Radio-resistant SiHa and ME180	*IER3*	Increased surviving fraction of a radio-resistant cells
miR-181a [87]	Up in resistant cells	Radio-resistant SiHa and ME180	*PRKCD*	Inhibition of apoptosis
miR-15a-3p [90]	Down in resistant cells and tissues	Radio-resistant HeLa and SiHa	*TPD52*	Increased proliferation and reduced apoptosis of cells exposed to the radiation
miR-132 [91]	Down in resistant cells and tissues	Radio-resistant HeLa, SiHa, and C33A	*BMI-1*	Increased proliferation and reduced apoptosis of cells exposed to the radiation
miR-145 [92]	n/a	HeLa, SiHa, and Caski	*HLTF*	Enhanced radiation-induced cell viability, reduction and apoptosis of cancer cells
miR-145 [93]	n/a	Tera cells	n/a	Induced radiosensitivity by reducing cell proliferation and promoting apoptosis
miR-499a-5p [53]	Down in cancer tissue and cells, overexpression enhance radiosensitivity of cells	HeLa and CaSki	*EIF4E*	Increased proliferation, invasion, and cell migration. Apoptosis inhibition
miR-4429 [94]	Down in resistant cells and tissues	Radio-resistant HeLa and SiHa	*RAD51*	Induce proliferation and DNA damage repair of cancer cells
miR-512-5p [95]	Down in resistant cells and tissues	Radio-resistant SiHa and radio-sensitive Me180	*MUC1*	Increase survival of cancer cells and decrease apoptosis
